# The Effects of Sensory Cues on Immersive Experiences for Fostering Technology-Assisted Sustainable Behavior: A Systematic Review

**DOI:** 10.3390/bs12100361

**Published:** 2022-09-27

**Authors:** Yaqi Zhang, Yao Song

**Affiliations:** 1 School of Design, Hong Kong Polytechnic University, Hong Kong SAR 999077, China; 2 College of Literature and Journalism, Sichuan University, Chengdu 610065, China; 3 Digital Convergence Laboratory of Chinese Cultural Inheritance and Global Communication, Sichuan University, Chengdu 610064, China

**Keywords:** sustainable game, sensory, immersion, behavior change

## Abstract

Games are consistently acknowledged as a powerful approach that can significantly impact people’s behavior towards living in a sustainable way. Sensory cues are regarded as influential factors in facilitating immersive experiences in gamified applications to foster sustainable behavior. As our perception of an environment is influenced not only by what we can see but also by additional sensory input such as sound and touch, additional sensory information can be part of the participant’s experience. This study systematically scrutinized game-based applications containing sensory cues to interpret current technology-assisted sustainable behavior development. This study provides a review of the impact of the sensory signals offered by video games, virtual reality, and augmented reality on pro-environmental behavioral intention. This research found that human senses can change the perception of immersion in multiple ways: visual (dimensions, angles, color), auditory (music, dialogue), and haptic, and these can affect sustainable behavior. Thus, we argue that multiple sensory modalities provide more opportunities to influence users to act sustainably. Based on the results, the theoretical contribution of this paper emphasizes the level of immersion, which is closely related to various sensory perceptions, and explains the correlation between them. In terms of industrial applications, it provides game designers, developers of VR and AR applications, and planners of sustainable education guidelines for the adoption of immersive scenarios.

## 1. Introduction

Climate change is considered to be the most urgent global issue of our time and is expected to generate a series of ecological, socioeconomic, and health impacts [1,2,3,4]. It has fueled a strong need for sustainable development [5]. In response to climate change, sustainable literacy and behavioral engagement are identified as effective mitigation strategies [6]. In order to enhance literacy acquisition and the quality of engagement, gamification is considered an effective approach [7], because it drives engagement and knowledge acquisition by applying game mechanisms in a non-gaming context to encourage users to adopt certain behaviors [8]. Therefore, many gaming applications, and much research, have established that gamification can mitigate the way human behavior affects climate change. Well-designed games have proved to be a more effective approach to promoting sustainable behavior than traditional slogans or incentive methods [9].

The scope of technology-assisted gamified applications discussed in this article includes virtual reality (VR), augmented reality (AR), mobile applications, 360-degree video, and video games. These emerging technologies intervene in gamified applications to provide diverse presentations of ecological scenarios and move towards bringing ecology closer to users through vivid experiences [10]. In these games, avatars, multiple identities, and the sense of embodiment reshape users’ ecological awareness and paradigms of interaction. This empathy and concern for the environment, as well as behavioral self-awareness, can motivate users to engage in more pro-environmental behaviors [11], such as reducing plastic consumption [12] and energy consumption [13], saving water [14], and optimizing recycling [15]. When users are exposed to this type of gaming application, they are actually exposed to a variety of communication cues as well as a richer media experience. These information cues potentially add depth to information processing which elicits the user’s willingness to invest a greater cognitive workload to effectively process eco-friendly information, which will ultimately help users to be persuaded towards the idea of sustainability [10]. In addition, sensory stimuli could reduce the cost of acquiring information, thereby shortening information processing and accelerating sustainable decision-making.

### 1.1. Defining the Game Concept

The concept of the game as used in this study includes both video games and gamification applications. In order to better understand the concepts of the games referred to in the following arguments, we first need to clarify the relevant terms for their definitions. “Video game” means a game that relies on an audio–visual apparatus with a narrative story, as defined by [16]. Another concept of the game in this research refers to gamification, mainly according to the definition given by [17]: “The use of game design elements in non-game contexts”. Our concept of the game is a combination of the two; hence, we take it to mean the use of game elements, thinking, and mechanics in games and in non-game contexts, particularly in a digital context [17,18].

### 1.2. The Connection between Gamification and Behavior

Gamification is closely related to human psychology, in particular, behaviorism [19]. Some researchers even define gamification in terms of behavioral science: “Gamification is a designed-behavior shift through playful experiences” [20]. Behavioral psychology works to understand how particular manipulatory environments can control and replicate certain behavior [21]. Therefore, the design of the game environment may have an impact on user engagement behavior [21]. Some researchers have explained the link between gamification and human psychology and behavioral science [22,23]. For example, in fundamental behaviorism, enforcing the desired behavior through rewards and correcting misbehavior through the denial of rewards, or punishment, is similar to rewarding and punishing mechanisms in gamification with the use of points and badges, or upgrading and demoting the player in a game environment [24]. It is believed that the real power of gamification is its ability to produce desired behavioral changes [25].

### 1.3. The Connection between the Immersiveness of Gaming and Psychology

There have been numerous critical arguments about the definition of immersion. One accepted definition interprets it as the complete involvement with something, or the feeling of being surrounded by it [26]. In the context of games, immersion is interpreted as a powerful play experience and a very important interactive experience. Gamers, designers, and games researchers have significant concerns about the impact of immersion on the player experience. Players experience different levels of involvement with a game: the highest level of involvement equals immersion [27]. The immersion experience of gamers is divided into three types of engagement: the lowest level of engagement means the players spend time, effort, and attention; the middle is engrossment, referring to players investing emotions in the game, and the highest level is total immersion, meaning when gamers totally cut themselves off from reality. Immersion also can be categorized into spatial and emotional dimensions. Spatial immersion refers to a virtual environment that can be achieved by deliberately controlling the scene. For example, quickly switching the angle of the spatial environment, zooming in or out, etc. The emotional aspect refers to the user’s emotional immersion, their involvement with the emotional ups and downs of the narrative [25].

Although these help us understand immersion, it is difficult to create a high-quality immersive experience because immersion is closely related to other psychological factors, especially flow, emotion, attention, empathy, and motivation [27]. Moreover, these psychological factors are also important variables affecting behavioral intention [28,29]. The level of immersion of a game affects a gamer’s feelings and emotions. The variability of emotion relates to presence, empathy, and atmosphere. In gamers’ experience, a lack of empathy makes it difficult for them to shift consciousness. Therefore, it is hard to achieve a sense of immersion [26,27]. Immersion can be seen to be closely related to Czsentmihalyi’s concept of “flow”, in which any distraction will make person feel that the experience of flow disappears [30]. Hence, the variation in flow also causes different forms of immersiveness. Attention, in an immersive gaming context, means that the player devotes all their attention and thoughts to the game rather than to the surrounding environment [31]. Attention is a necessary element of achieving total immersion [27]. In addition, attention can be divided into “focus”, “locus”, and “sensus” [32] Attention affects the experience of gaming immersion. It is hard to archive immersion without the component of attention [33]. Any changes in the game will affect the player’s attention and therefore their engagement with the game. Emotional immersion does not allow users to feel a “physical presence” in the scene, but instead enables them to cognitively and emotionally resonate with the virtual world [34].

### 1.4. Research Question and Current Study

Gaming is confirmed as a highly effective approach to motivating people toward sustainable awareness [35]. From reviewing earlier works, it was seen that digital gaming approaches could be applied to climate change resilience [6,36,37], waste management, [38] wastewater management [39,40], environmental fundraising [41], and energy conservation [42,43]. The use of digital gaming for environmental education in issues of sustainable behavior resulting from environmental literacy mainly focuses on knowledge-sharing, the cultivation of competencies, and responsible behavior.

Current research trends show that sensory cues stimulated by an immersive virtual experience can mediate behavior in the context of the gamification of sustainability awareness. The virtual sensory cues, or stimuli, can be divided into three features: visual, auditory, and haptic. As Masuch signified, visual features refer to dimensionality, perspective, color, realism, and presentation [44]. Auditory stimuli are music and dialogue. Haptics offers a tactile sense of feeling in a virtual environment.

While there has been significant research on the variables and empirical experiments conducted on sustainability-themed gamification, the multisensory immersive experience of such games has not been thoroughly accounted for and summarized [9]. Immersion in the game and behavior change are impossible to achieve without the experience of psychological elements such as flow, attention, emotion, and motivation. Scholars have recently carried out relevant research on the elements of immersion enabled by these senses. This study, therefore, provides an overview of the sensory factors that influence behavioral change in a pro-environmental context. Hence, the research questions posed in this study are the following: How do different senses promote sustainable behavior within an immersive experience? How can an immersive gaming experience trigger sustainable user behavior?

Based on the questions raised above, we conducted a literature review and discuss the findings. The arrangement of the article is organized as follows: in Section 2, we elucidate the methodology used in the study and the sources of data collection. In Section 3, we list the findings of the result and present the research trends. The discussion of the results occurs in the following part, Section 4, analyzing how sensory cues and immersion promote changes towards behavior that are more environmentally sustainable, and which factors might be responsible for this. Section 5 is a summary of the article and a discussion of the implications and the limitation.

Currently, mainstream research on sustainable behavior adoption includes design for sustainable behavior, eco-technology, and research into VR and sustainable behavior. In technology-assisted sustainable gamification research, the existing theories revealed are hedonic, utilitarian, embodiment, motivation, novelty, vividness, and cognition of engagement performance [12,15,45,46]. Therefore, the theoretical contribution of this paper emphasizes the level of immersion, which is closely related to various sensory perceptions, and explains the correlation between them. On the other hand, in terms of its contribution to industrial applications, it provides game designers, developers of VR and AR applications, and planners of sustainable education with guidelines for the adoption of immersive scenarios with visual (dimension, perspective, color), auditory (music, dialogue), and haptics as a tool to enhance sustainable behavior and performance.

## 2. Methodology

### 2.1. Systematic Review Method

A thorough, replicable, and transparent assessment of the vast body of literature on complex subjects such as gamification can be provided through a systematic review. It gives a general sense of the quantity, type, and caliber of the available evidence in relation to the main research issue. The systematic method could thus support the development of substantial and comprehensive implications for theory and future study [23]. The systematic method involves an exhaustive search of all the available data on a topic. In this case, the primary sources for review were collected from databases and multiple sources. The main objective was to identify the sources on sensory-cues-induced immersive games for sustainable behavioral change. A keyword string search was used to interrogate the databases by title, abstract, and author, and considered all articles published up to March 2021.

### 2.2. Criteria and Objective

#### 2.2.1. Screening Strategy and Inclusion Criteria

According to the ROSES criteria proposed by [47], the screening process is divided into three steps: title screening, abstract screening, and full-text screening. To ensure research quality, only peer-reviewed journal articles and conference papers were included in the final selection. Book chapters, non-peer-reviewed journal articles, and grey literature were excluded. The reason for the inclusion of conference papers is that discussing game interaction constitutes a large proportion of computer science and human–computer interaction research [48]. In addition, identifying articles from conference proceedings is generally considered good systematic review practice [49]. Articles reviewed include only those written in English.

#### 2.2.2. Inclusion and Exclusion Criteria Standards

Furthermore, the criteria for inclusion of studies were that they should involve a sensory cue or immersive game to drive users to adopt sustainable behavior, including learning behavior, participate in the construction of these, and aim to encourage the adoption of environmental climate change behavior. In addition, articles were selected that discussed the immersive experience of games with the theme of sustainability that aimed to change the user’s psychological cognition and behavioral intentions. However, articles that addressed the topic of eco-friendly games but that did not involve behavioral or experience change were excluded from the screening process. The literature screening results are summarized in Table 1.

#### 2.2.3. Assessment of Quality

The quality of selected studies was independently confirmed by two researchers (Y.Z. and Y.S.) via the Joanna Briggs Institute Critical Appraisal Checklist for Studies Reporting Prevalence Data [50]. Following the suggestions mentioned above, the response of “yes” counts as one point: a total score of greater than six was regarded as being of good quality and was included accordingly.

### 2.3. Search Strategy

We carried out Boolean keyword searches in three main databases, including the Web of Science, Scopus, and ACM Digital Library, based on the combining the terms “visual” OR “auditory” OR “haptics” OR “immersive” with “sustainable”, “pro-environmental”, “behavior”, and “game” as keywords to create different searches. The electronic database search was carried out on 18 March 2022. The database search results are listed in the following tables.

### 2.4. Search Results

**Figure 1:**
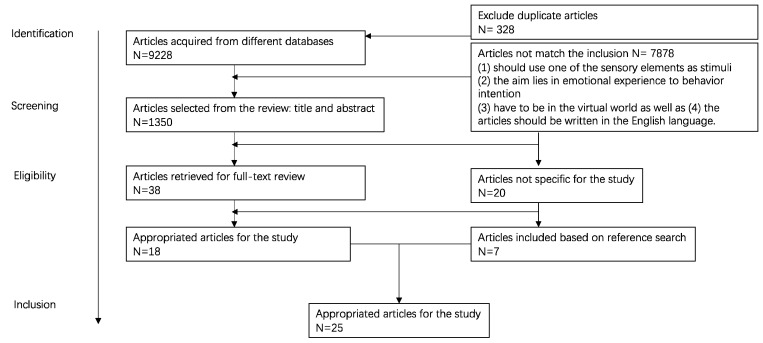
Flow chart of the systematic review process.

This systematic review process is shown in Figure 1. After a comprehensive search was conducted, the initial selection was made from around 9228 articles from the three databases, as shown in Table 1. A screening process for duplication was conducted, a total of (n = 328) articles were removed, and a total of (n = 8900) articles were retained. At this stage, we manually scanned the titles and abstracts of these filtered articles to check their relevance to the inclusion criteria, which resulted in a list of 38 articles. After the initial selection and exclusion process, we carefully read the full text of these remaining papers. In the end, 25 articles were identified as related to the theme, including 7 references to research projects. The results of these searches are summarized in the table below.

## 3. Findings and Results

**Figure 2:**
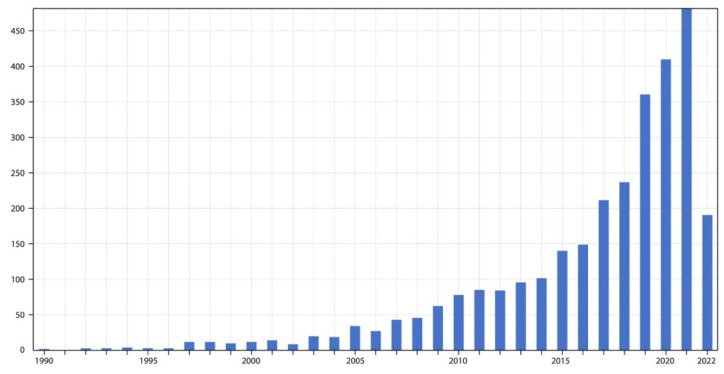
Research trends in volumes of publication from 1990–2022 from Web of Science database.

From the extant literature, a trend can be seen to emerge regarding the need to understand the effect of the characteristics of gaming on behavioral intention. We can see from Figure 2 that there has been significant research on the impact of immersion on behavioral intention towards sustainability in games. It facilitated our study to summarize these findings and the effects of an immersive experience, thus offering a better understanding of pro-sustainability behaviors in gamification. After scrutinizing the online literature on behavior change towards sustainability, the sensory traits were summarized, as shown in Table 2. The table shows a list of empirical studies and the main findings on different forms of sensory stimulation and immersiveness as stimuli for pro-environmental behavior change. The results are analyzed below.

### 3.1. Visual Applications for Immersion

#### 3.1.1. Dimension

The sophistication of visual imaging is closely related to the feasibility of an immersive environment. In terms of visual stimuli, dimension can play an indispensable role in influencing sustainable action. For example, a pilot study in sustainable urban design was proposed by Isaacs et al. It investigated an immersive 3D virtual world that can be used as a tool to support decision-making that successfully addresses spatial and temporal issues via real-time 3D rendering. This also changes the decision-making process by offering stakeholders better and equal accessibility to sustainable activities [60]. Earlier research suggested that 3D rendering leads to strong psychological responses. Janicke and Ellis indicated that 3D rendering has a significant capacity to retain people’s attention and heighten emotional arousal [61]. In addition, lifelike 3D models provide realistic visualization support for environmental education [62]. Three-dimensional visualization as visual information has a distinct impact on understanding the problems associated with resource depletion compared with 2D visualization [14]. Therefore, the dimensional aspect of visual stimuli could be important for strengthening immersiveness and promoting sustainable behavior. The latest study to explore the effect of combining a real-time 3D avatar with physical activities produced the finding that a realistic visual appearance and physical interaction will make the gaming experience more immersive. Visual sophistication and complexity are related to the player’s level of immersion, which can lead to a better experience [8].

#### 3.1.2. Style

A realistic aesthetic style as a visual stimulus in the game environment potentially has an impact on pro-sustainability behavior. The extant literature suggests that a realistic style would have a significant influence on motivation [63]. Segaran and his colleagues [64] discussed the effects of realism on triggering motivation in game-based learning. Moreover, Baylor claimed that realistic virtual avatars can mediate the enjoyment and motivation experienced in a game, creating self-efficacy, which leads to behavior change [64]. From the perspective of motivation to that of behavior adoption, the social cognitive theory argues that individuals with high environmental self-efficacy would have higher expectations, so they will be involved in more sustainable behaviors [65]. Therefore, a realistic style of imagery is likely to have a positive impact on sustainable behavior.

#### 3.1.3. Perspective

Considering the element of perspective as a powerful visual stimulus in the gaming context means that it may have a strong impact on engagement with pro-sustainability behavior, which relies on a particular level of immersiveness. Denisova and Cairns examined the effect of first- and third-person perspectives, showing that more immersive feelings are experienced from the first-person point of view [66]. The angle of view was researched further, finding that immersive natural video content strengthens the feeling of spatial presence and a commitment to the natural environment, enhancing a connection with nature and thus enhancing pro-environmental behavior [67]. Additionally, some scholars have argued that an immersive system is a good way of encouraging motivation, awareness, and information transformation to evoke sustainable behavior [68].

#### 3.1.4. Color

Color appears to be an influential visual stimulus in enabling participation in sustainable behavior. The authors of [69] claimed that chromatically-related factors such as brightness, saturation, and intensity would prompt emotional effects in terms of individuals’ perception. Meanwhile, there is a strong relationship between color diversity and emotion. However, emotion is indispensably connected with people’s judgment and thinking, so it offers an insight into the emotional capability to motivate action and improve communication about climate change. It has been concluded that evoking emotions can optimally promote sustainable behavior change [70]. Correspondingly, the use of color in design is potentially associated with sustainable behavior.

### 3.2. Haptic Applications for Immersion

Innovative VR technology is widely used to explore education in sustainability, and it allows users to touch and feel virtual objects. Haptics interface interaction that works by receiving tactile and force feedback is regarded as a powerful sense with which to mediate the degree of realism [67]. Tactility can reveal hardness, softness, and texture. In terms of haptic stimuli, the feeling of touch can be considered an important factor in the game experience to facilitate sustainable action.

Cho et al. proved that the intervention of force feedback in haptic gaming provides a more immersive environment to stimulate a higher level of engagement [33]. Earlier scholars demonstrated that immersive VR games caused significant changes in cognition and behavior intention. Furthermore, within the context of sustainability, it has been proved that adopting a virtual reality approach is effective in increasing awareness of environmental threats and encouraging sustainable behavior [71]. From these previous studies, we can infer that the intervention of the haptic sense would contribute to sustainable action.

The virtual experience is created in an immersive environment that exposes individuals to vivid information with immediate feedback, bringing significant changes to their cognition and behavior. For example, in a VR environment experiment (N = 156 testers) relating to water conservation, the results showed that it enhanced pro-sustainability behavior [45]. From this, we can conclude that enabling user engagement can contribute to persuasive technology in a pro-environment context.

### 3.3. Auditory Sense for Immersion

#### 3.3.1. Music Applications to Enhance Attention

Music can be seen as an important stimulus for behavior change. Earlier research proved that music is increasingly used to facilitate task engagement and positively impact task performance in a gaming context [72,73]. Bugter and Carden demonstrated that different types of music have different effects on concentration and attention performance [70,74]. People find it easier to concentrate and focus their attention when they are exposed to self-selected music. Attention is a significant determinant of human perception and constructions of reality; therefore, any variation in attention will considerably influence people’s actions [75].

#### 3.3.2. Dialogue and Immersive Experience

Typically, dialogue is presented in games in two ways, orally and as a written text. Many relevant studies agree that the oral and written dialogue in the game directly enhances the gaming experience. Dialogue can create an immersive experience, especially in role-playing games [76,77]. Empirical research was carried out to demonstrate that emotions will be produced even from dialogue that is written: therefore, dialogue and verbal or written communication would be likely to elicit emotions and change behavior [78]. Single-player and multiplayer game participants experience different kinds of dialogue approaches differently. These approaches to dialogue exist on two levels: one relates to ‘direct ludic communication’ (giving commands or enforcing rules within the game’s system) and the other is defined as ‘diegetic communication’ (deep dialogue that branches out and extends). Recent trends imply that for full immersion in a game’s fictional world, it is more effective to disguise the ludic communication by shifting it to a diegetic level: this constitutes a significant strategy of persuasion [77]. A semantic message is regarded as an impactful stimulus for behavior in human-computer interaction, and it has been demonstrated that this strategy of persuasion is equally effective via verbal messages in the virtual world [79].

### 3.4. Persuasion Strategy

In relation to persuasive technologies, Cialdini and James identified several ways through which the act of persuasion can be carried out: authority, commitment, likeability, reciprocity, and scarcity [80].

#### 3.4.1. Authority

The “authority” approach for persuasion is based on the fact that “people can be persuaded by experts or persons who have authority”. Sending a persuasive message based on authority can also be described as commanding, convincing, and suggesting. More specifically, “commanding” implies a new obligation for the individual; “convincing” refers to an established goal requiring particular desired behavior; “suggesting” represents providing information so that the individual can decide for themselves to change their behavior [80]. In relation to authority, Saunderson and Nejat suggested that robot interaction with humans should avoid exploiting the “authority” approach [81]. Their findings showed that persuasion by using a role of authority produced a low success rate compared to that which adopts a peer role. Research by Zalake et al. on the authority attribute also demonstrated that people have the lowest likelihood of intending to return to robot interaction if it adopts an authority role [80].

#### 3.4.2. Likeability

In contrast, the likeability attribute gained a high score in terms of persuasion. Likeability succeeds in persuasion by being associated with the kind of people that the user likes [82]. Likeability is triggered by interpersonal similarity: people are more likely to perceive themselves as closer to someone else if they are similar to them than if they are dissimilar [83]. Previous work found that similarity can evoke and increase the sense of liking in the context of persuasion [84].

#### 3.4.3. Commitment

In terms of commitment as a principle of persuasion, the research of Oinas-Kukkonen et al. implies that hedonic value is governed by commitment [85,86]. Helmefalk and Rosenlund asserted that various cognitive and affective responses are impacted by a hedonic perspective [15]. Hedonic value is an individual perception of fun and playfulness which is focused on improving an individual’s feelings and reducing effort, as well as influencing attitudes and behavior [87]. Thus, a hedonic attitude significantly impacts an individual’s “green” behavior [88]. Moreover, Mickaël proved that commitment plays an important and effective role in motivating pro-environment behavior [89].

#### 3.4.4. Reciprocity

Reciprocity refers to more than material favors or gifts [90]. The value of reciprocity consists of instrumental value and symbolic value. The instrumental value of exchange governs behavioral preferences, whereas the symbolic value of constant reciprocity substantially influences sentiments of trust, affective respect, and solidarity.

#### 3.4.5. Scarcity

In terms of scarcity, Cialdini observed that “Whatever is rare, uncommon or dwindling in availability—this idea of scarcity—confers value on objects, or even relationships” [90]. Shen identified scarcity as a heuristic cue that directly impacts the way things are evaluated [91]. Hamilton et al. revealed that different types of scarcity will influence individuals’ decision-making at different stages of the journey towards a decision [92]. Arroyo and others found that adopting the scarcity principle with design intervention that involves eco-feedback in persuasive technology can obtain positive behavior change towards sustainability [90,93].

## 4. Discussion

In this paper, we argue that novel technology-assisted gamification applications can provide sensory stimulation that has the potential to enhance the options for adopting sustainable behaviors. By reviewing the existing literature related to sensory stimulation and immersion, we propose potential interactions between multisensory modalities (visual, auditory, and haptic) in sustainable gamification applications, which may lead to pro-environmental behaviors, sustainable decision-making, and green consumer choices. The sensory element of transforming sustainable behavior through immersion is crucial in the gamification process. In the context of developing sustainable behavior, cognition and behavior associated with understanding climate change, environmental protection, and environmental knowledge learning through games/gamification will be different from the cognition involved in non-game intervention. Matching suitable sensory stimuli to specific scenarios will be an important consideration in the future. We hope that this systematic literature review serves to identify the need for greater emphasis on immersion in research into the gamification of sustainable awareness and behavior. Armed with this knowledge, those planning sustainable behavioral interventions can select proven methods and experiences of specific situations for practical applications.

### 4.1. Sensory Cues Help an Understanding of Environmental Issues

There are many environmental problems that cannot be realized because they are difficult to address; this is why simulations are important in constructing such experiences, to bring people closer to the problems and help them to identify with the seriousness of environmental issues in a playful way. These gamified simulations need to engage the senses to arouse people’s awareness because some environmental issues are not obvious. The following are several scenarios that were taken as examples. In the first scenario, most environmental issues are invisible to the naked eye, such as Co2 emission. The lack of awareness of this means that people have difficulty connecting environmental pollution with their everyday behavior. In the second scenario, phenomena associated with environmental degradation and natural disasters often happen far away geographically, so people have difficulty connecting future negative consequences to current behavior. Third, the increasing human physical disconnect from nature leads to a loss of emotional attachment to the environment [71]. Therefore, emerging digital technology such as virtual reality (VR) and augmented reality (AR), which involve multisensory (visual, auditory, and haptic) stimulation, is seen as a promising tool to convey vivid information and create immersive experiences, offering a narrative of environmental issues to provoke a more emotionally attached response. Other relevant research also agreed that a multisensory experience provides greater opportunities for encouraging sustainable behavior [10,94,95].

### 4.2. Sensory Cues Shape the Level of Immersiveness 

Sensory cues bring environmental issues closer, which is inseparable from the impact of perception on immersion [96]. The different sensory settings of sustainable gamification enable a range of different levels of immersiveness, from engagement to emotional attachment and then total immersion [97,98]. Immersive scenarios prompt the perception of higher positive emotions and a sense of presence compared with non-immersive experiences [99]. Immersion and emotions of positive affect are considered to be the most important components of experience in developing climate resilience and sustainability awareness [36,100]. Additionally, the higher the level of immersion, the easier it is to stimulate environmental awareness and empathy [11]. The existing research also revealed that immersive experience and emotional enjoyment tend to have a significant impact on users’ behavioral intentions towards sustainability [9]. Therefore, there is an increasing trend for gamification to be applied, employing sensory feeling to enable immersive experiences to solve environmental problems.

### 4.3. The Sensory Impact on Cognition

It is clear that immersion affects visual engagement. The influence of visual-related elements, in general, still occupies the largest proportion [14,100]. Much immersion is created by visual perception, which not only affects mood, cognitive workload, and sense of presence but also affects temporal, spatial, and risk perception in the sustainable context for three reasons: First, different perspectives lead to various information processing. Experiencing the first-person and third-person perspectives in avatars can produce different perceptions because previous research has shown that the brain processes information differently when viewing tasks from different perspectives [101]. Thus, people might feel a stronger connection to their avatar when experiencing it from the first-person perspective [102]. Secondly, realism influence immersion. Participants in the virtual environment were found to perceive a higher sense of spatial presence to enhance emotional response and shorter perception of the temporal distance than those in the video condition [103]. Therefore, they felt greater engagement in the virtual environment than in the video condition [46,104]. Thirdly, Bailey and his colleagues showed that the vividness in visual information requires a lower cognitive load than the text condition, even if the image quality is not particularly advanced [105]. It was also able to elicit pro-environmental behaviors that changed participants’ actual energy use [105]. Visual information influences affective valence. In addition, Geslin and his colleagues concluded that there is a significant correlation between chromatic diversity (brightness, saturation, and hue) and emotion (joy, sadness, fear) in in-game visual information [69]. When there are sources of different colors, the neuronal activity associated with the observed behavior may increase [106]. A high level of color diversity can also generate positive emotions [69]. Furthermore, Ham and Midden found that visual image feedback utilizes less cognitive resources than textual feedback, which requires more mental effort [107]. Chirico et. al found that when presented with a more vivid way of representing [12], participants felt more of the illusion of being there (presence) in the simulated environment, supporting the antecedent effect of vividness on the presence [108].

The sensory-rich experiences in the virtual reality led to a greater feeling of being present and interconnection between the self and nature compared to the video. Immersion into the natural environment can lead to an awareness of looming environmental risks and a greater understanding of nature [46]. Increased connection to nature after exposure to virtual nature may not always translate into more pro-environmental behaviors. There are other factors that have not been explored, such as the duration of exposure and the type and familiarity of the natural environment [67].

### 4.4. Immersion Influences Behavior Intention

The immersive experience is strongly associated with sensory and focused attention [109]. Fauville et al. defined immersion as “a psychological state characterized by perceiving oneself to be enveloped by, included in, and interacting with an environment that provides a continuous stream of stimuli and experiences” [71]. When individuals experience greater immersiveness, they may show greater behavioral intention [109]. More importantly, behavioral intention is not directly impacted by technology-related stimuli but is mediated by immersion. Thus, immersion is considered a key determinant in promoting the adoption of sustainable behavior [110]. 

Designers of gamified interventions must consider the effect of participants’ sensory information on behavior. Users had higher self-efficacy when they believed that positive environmental changes in the game depended on their behavior. Virtual environment games not only shape participants’ experience and perception of the virtual world, but they also have the potential to shape their attitudes and behavior outside the virtual world. There are two types of pro-environmental behavior that occur in a variety of game scenarios. One is related to the context of daily life. For example, research is most often aimed at reducing water, plastic, and paper consumption. Another is the embodiment of a new role, which enhances the user’s environmental awareness by changing participants’ roles and integrating them into environments that are geographically distant, and not easily accessed in everyday life. These experiences foster the interconnectedness between nature and the self and will lead to more merging of the self with nature and pro-environment behavior [46].

### 4.5. Immersive Gamification Facilitates Behavior Change 

The use of immersion tends to evoke a positive emotion, so it can determine behavioral performance [37,111]. In particular, immersive persuasion can increase the sense of pleasure experienced and thus can more effectively motivate behavior change. The advantages of immersive games as a tool for behavior change are that individual motivation can be stimulated through emotional involvement, embodied interaction [40,112], and an immersive experience, combined with game mechanisms such as competition rankings and rewards to enhance motivation [40,113]. In this way, users are likely to change their attitude toward sustainable participation and achieve the goal of behavior change [103,114,115,116].

In most studies, it was unclear how long the effects of gamification on behavioral change lasted. This is a common limitation in studies and is worth highlighting and addressing in future work. With regard to sustainable gamification, user information retention is also an issue (see [117]). We do not yet know how much environmental literacy and types of information are retained by users and implemented in action, but it is promising that, according to the evidence, immersive VR can improve retention over time: for instance, students retain knowledge of marine conservation after a few weeks of underwater experience [116]. Moreover, it is unclear how immersive VR/AR games compare with other mobile games in information retention, a question that awaits exploration [118]. Most of the behavioral changes discussed in the studies focused on one behavior and did not discuss the improvement of pertinent pro-environmental behaviors after awareness was enhanced through gamification. In addition, in the articles reviewed so far, most of the research subjects were young adults, adolescents, and children. However, these are not representative of the wider population. For future research, it is important to focus on participant demographics, as age, gender, experience with technology and gaming, attitudes, and lifestyle may all have an impact on the results.

### 4.6. Limitation

We can only rely on the authors’ textual descriptions of game applications and their usage, so there is a limitation where the paper does not fully cover the context. The game technologies selected in our current research include traditional digital video games and games with emerging VR/AR technology. The gaming immersion they create will be different because of the difference in techniques and visual presentation formats. While VR technology tends to offer greater visual immersion, digital video games can also create immersion through narrative stories. Therefore, the level of immersion cannot be judged entirely by the level of technology. Thereby, the choice of technology should be based on different purposes and scenarios, as well as personal preference. In addition, the type of game and participants’ demographic context might also influence behavioral intentions. Hwang et al. identified that the effect of gender could affect students’ attitudes and behavioral intentions [119]. A future study is needed to review the effect from this perspective. Last, since the current paper focuses on the general effect of sensory cues on immersive experiences to foster sustainable behavior, some of the sources [45,46,69,103,105] are not empirical studies. Thus, a future study would be based on the current conclusions and a meta-analysis, conducted later, which would include results pooling, publishing bias detection, and heterogeneity and sensitivity analysis for a thorough investigation.

## 5. Conclusions

With the advancement of technology, the current trend is towards a focus on exploiting advanced information technology such as VR and AR in heuristic training for climate change awareness, using vivid information technology to promote involvement. Bringing humans close to nature through immersive scenarios helps them understand environmental complexity, causes and effects in nature, and even embodiment, “standing in the shoes of others” to feel that their species is part of wider nature. The gamification of new technology interventions may change the way we behave sustainably. These applications can provide users with a multisensory experience, in contrast to traditional single-sensory stimulation. 

However, few attempts have explored the relationship between immersion and sustainable behavior. In order to address this research gap, this study utilized a systematic review method to indicate that human senses can change the perception of immersion in multiple ways: visual (dimensions, angles, color), auditory (music, dialogue), and haptic, and these can affect sustainable behavior. Thus, we argue that multiple sensory modalities provide more opportunities to influence users to act sustainably. Based on the results, the theoretical contribution of this paper emphasizes that the level of immersion, which is closely related to various sensory perceptions, explains the correlation between them. On the other hand, in terms of its contribution to industrial applications, it provides game designers, developers of VR and AR applications, and planners of educational programs on sustainability with guidelines for the adoption of immersive scenarios with visual (dimension, perspective, and color), auditory (music and dialogue), and haptic elements as a tool to enhance sustainable behavior and performance.

## Figures and Tables

**Table 1:** Total number of articles searched from different databases.

Database	Search Terms	Hits
Web of Science	Immersive sustainable gamification contained (“virtual” OR “auditory” OR “haptics” OR “immersive”) mixed combined with “sustainable”, “virtual”, “game” OR “gamification” as keywords to make different combinations.	2977
Scopus		5099
ACM		1242

**Table 2:** Summary of the sensory traits of immersion in the literature.

Authors	Purpose of Study	Result (Factors Impact on Environmental Behavior)	Location	Sample	Technique	Measure
Geslin et al., 2016	To identify a co-relationship between chromatic environment (color perception) and emotional effect.	Chromatic stimuli have a strong correlation with emotion.	France	85		Semantic subjective questionnaire.
Bailey et al., 2015	To investigate the effect of vivid messaging on energy-saving behavior.	The use of vividness would effectively leverage pro-environment behavior.	USA	70	Two-way covariance (ANCOVA)	2 × 2 between-subjects experiment.
Ahn et al., 2016	To test how environmental involvement is increased through sensory experience.	Sensory-rich experiences and the interconnection with nature can elicit the perception of environmental risk.	USA	228		3 experiments.
Ahn et al., 2014	To test the effects of short-term immersive experience on environmental behavior.	People who had immersive experience changed behavior by reducing paper consumption.	USA	107	ANCOVA	Experiments.
Fonseca and Kraus, 2016	To identify how immersion, presence, and narrative affect pro-environmental attitudes and behaviors.	Immersion and emotional impact considerably enhanced a pro-environmental attitude.	Denmark	64		A between-group design experiment.
Soliman et al., 2017	To examine the relevance of nature videos to pro-environmental behavior.	Viewing immersive nature-related content can increase a connection with nature but not pro-environmental behavior.	Canada	230	ANOVAs	2 × 2 between-subjects experiment.
Hsu et al., 2018	To test the relationship of vivid information with sustainable behavioral intention.	Use of immersion in games can cause a significant change in cognition and behavior intention towards sustainability.	Canada	165	ANOVA	2 × 2 between-subjects.
Markowitz et al., 2018	To test the effectiveness of immersive VR on climate change from different points of view.	The immersive embodiment experience resulted in a more positive attitude toward the environment.	USA	47	ANOVA	Experiments and 488 people for field study.
Chirico et al., 2021	To compare the impact of concrete and numerical information on pro-environmental behavior.	More “concrete” vivid information has a stronger persuasive ability to promote pro-environmental behavior.	Italy	60	One-way ANOVA	172 surveys, 60 experiments.
Breves and Heber, 2020	To examine whether the immersive video can influence a commitment to nature.	Immersive videos provide a stronger feeling about and commitment to the environment.	Germany	56	One-way ANOVA	A 2 × 1 between-subjects experiment.
Larson and Edsall, 2010	To examine the perception of different dimensions in visual information.	Dimension as visual information has a distinct impact on understanding the problem of water resources.	USA	76		A quasi-experimental approach.
Fox et al., 2020	To test the effects of gaming in encouraging individuals to undertake environmental improvement.	Greater perception of environmental risk leads to environmental behavior.	USA	190		Experiment.
Schroth et al., 2015 [51]	To evaluate the impacts of different presentation formats in climate change planning.	Visualization directly contributes to increased climate awareness and understanding.	United Kingdom	46	Paired-samples t-test	Questionnaire.
Hartmann and Apaolaza-Ibáñez, 2008 [52]	To test whether virtual experiences of nature increase individuals’ perception.	Virtual immersive environmental experience has a distinct influence on environmental attitude.	Spain	432		Interview.
Klein and Hilbig, 2018 [53]	To identify whether the connection with nature can foster pro-environmental behavior.	Exposure to natural, immersive experiences can encourage pro-environmental behavior.	Germany	120		2 experiments.
Desnoyers-Stewart et al., 2019 [54]	To examine the effect of breathing techniques in arousing psychological synchronization.	These bio-senses can encourage self-awareness and connectedness to a pro-environmental attitude.	Canada			
Pimentel et al., 2019 [55]	To allow users to experience environmental degradation to encourage pro-environmental behavior.	The immersive experience may contribute to mitigating the effects of climate change.	USA			
Shevchuk and Oinas-Kukkonen, 2020 [56]	To explore immersive and non-immersive user experiences.	Immersion can help developers in the decision-making process of choosing technological approaches.	Finland	50		Between-subjects experiment.
Pietra et al., 2021	To present how sensory feedback is a driver for eco-sustainable behavior.	Haptic and visual feedback positively impact a reduction in energy consumption.	Italy	10	ANOVAs	Experiment questionnaires.
Lu and Liu, 2015 [57]	To explore the impact of 3D AR immersive applications on behavior change.	Ecologically-based games improved participants’ confidence and engagement level.	Taipei	51	ANCOVA	Questionnaire and experiment.
Veronica and Calvano, 2020	To understand how games can be used effectively to promote sustainable behavior.	Participants are able to change behavior through ecologically-based games.	Italy	50		Experiment.
Fang and Sun, 2016	To compare animated and static eco-visualization for sustainable behavior.	Animated imagery evoked more emotional valence and greater sustainable behavior intentions than a numeric interface.	Taiwan	93	Single-factor ANOVA, regression analysis, correlation analysis	Questionnaire.
Esmaeili and Thwaites, 2021 [58]	To attempt to use a VR game about recycling to create an undesirable environmental experience to address the issue.	VR experience becomes a possibility to address environmental issues.	Malaysia			Pilot study.
Vasey et al., 2019 [59]	To implement narrative and gameplay mechanisms to discourage plastic usage.	Immersive technology raises microplastic awareness.	USA			
Isaacs et al., 2011	To evaluate 3D interactive information about sustainability, providing an immersive experience for users.	3D information enhances the user’s perceptual and spatial capabilities to process information.	United Kingdom			

## Data Availability

The data used in this study are available upon request from the corresponding author.
